# Supplementary material for: HRDE-2 drives small RNA specificity for the nuclear Argonaute protein HRDE-1
Source: Nat Commun. 2024 Feb 1;15:957. doi: 10.1038/s41467-024-45245-8 (PMC10834429; doi:10.1038/s41467-024-45245-8)

Supplementary Figures

HRDE-2 drives small RNA specificity for the nuclear Argonaute protein HRDE-1

Shihui Chen and Carolyn M. Phillips

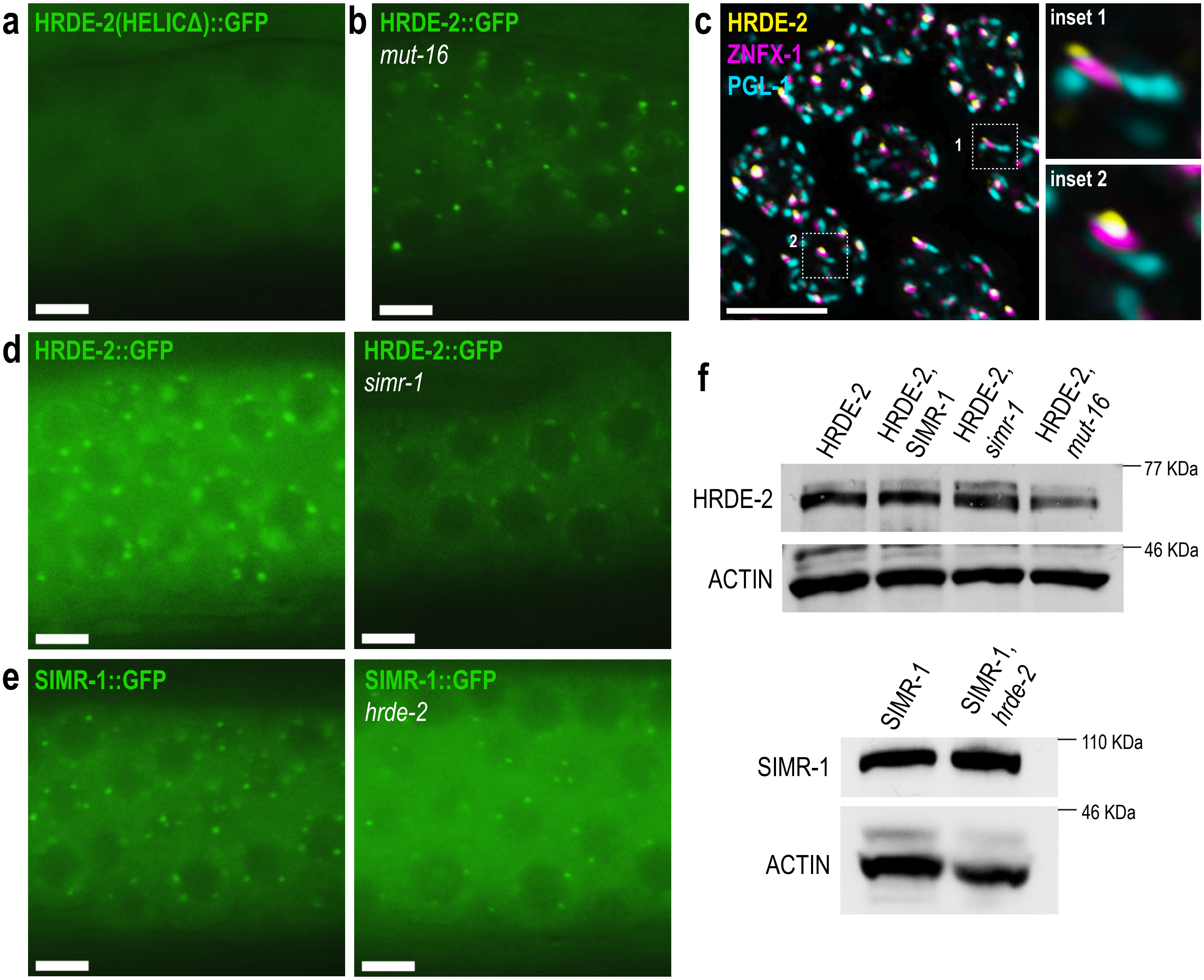

Supplementary Fig. 1 Expression and localization of HRDE-2 and SIMR-1. a Live imaging of HRDE-2(HELICΔ)::2xTy1::GFP in a day-one adult germline. At least five individual germlines were imaged. Scale bar, 5 μM. **b** Live imaging of HRDE-2::2xTy1::GFP in a day-one adult, *mut-16* mutant germline. At least ten individual germlines were imaged. Scale bar, 5 μM. **c** Immunofluorescence imaging of a HA::tagRFP::ZNFX-1; HRDE-2::2xTy1::GFP dissected day-one adult germline, using antibodies against HA, GFP, and PGL-1. All images are 3D projections following deconvolution. At least eight individual germlines were imaged. Scale bars, 5 μM. **d** Live imaging of HRDE-2::2xTy1::GFP in wild-type (left) and *simr-1* (right) day-one adults. HRDE-2 localization to germ granules is modestly reduced in a *simr-1* mutant. Images were taken using the same exposure time and processed identically. At least five individual germlines were imaged for each genotype. Scale bars, 5 μM. **e** Live imaging of SIMR-1::GFP::3xFLAG in wild-type (left) and *hrde-2* (right) day-one adults. Images were taken using the same exposure and processed identically. At least five individual germlines were imaged for each genotype. Scale bars, 5 μM. **f** Western blot of HRDE-2 protein level in wild-type, *simr-1*, and *mut-16* day-one adults (top), and SIMR-1 protein level in wild-type and *hrde-2* day-one adults (bottom). Anti-Ty1, anti-HA, and anti-Actin antibodies were used for HRDE-2, SIMR-1, and Actin respectively.

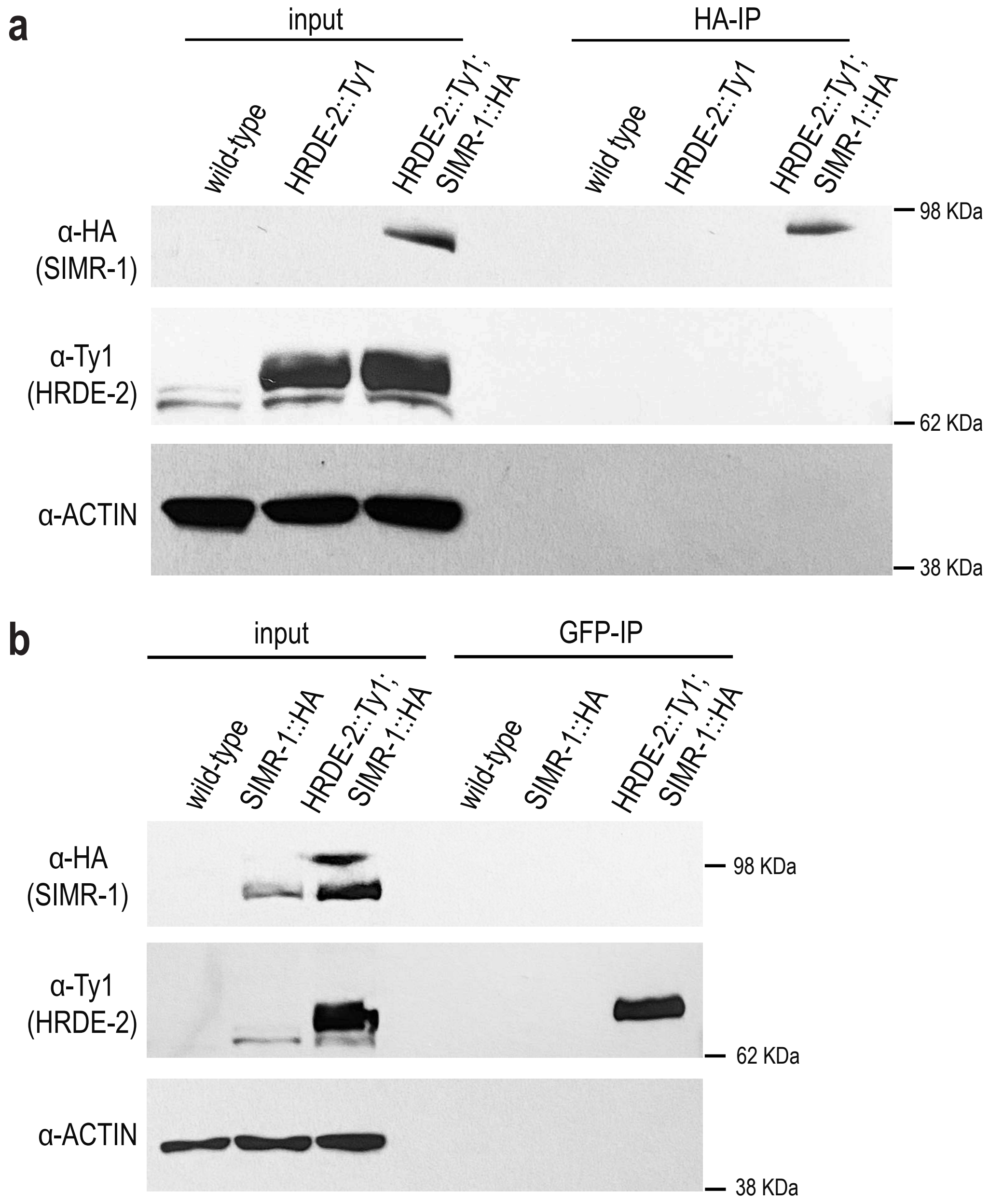

c

Peptide counts from IP-mass spec in replicate 2 and 3

**Protein Name**	**wild-type IP**		**SIMR-1 IP**		**HRDE-2 IP**	
	rep 2	rep 3	rep 2	rep 3	rep 2	rep 3
SIMR-1	0	0	92	94	0	0
HRDE-2	0	0	0	0	15	13
HRDE-1	0	0	0	0	29	5
NRDE-3	0	0	2	0	0	0

Supplementary Fig. 2 SIMR-1 and HRDE-2 interaction cannot be detected by co-IP. a Western blot following SIMR-1 immunoprecipitation from SIMR-1::mCherry::2xHA; HRDE-2::2xTy1::GFP did not detect an interaction between SIMR-1 and HRDE-2. Wild-type and HRDE-2::2xTy1::GFP strains were used as negative controls. Anti-HA conjugated beads were used to immunoprecipitate SIMR-1::mCherry::2xHA. Anti-HA, anti-Ty1, and anti-Actin antibodies were used to detect SIMR-1, HRDE-2, and Actin, respectively. **b** Western blot following HRDE-2 immunoprecipitation from SIMR-1::mCherry::2xHA; HRDE-2::2xTy1::GFP did not detect an interaction between SIMR-1 and HRDE-2 . Wild-type and SIMR-1::mCherry::2xHA strains were used as negative controls. Anti-GFP conjugated beads were used to immunoprecipitate HRDE-2::2xTy1::GFP. Anti-HA, anti-Ty1, and anti-Actin antibodies were used to detect SIMR-1, HRDE-2, and Actin, respectively. **c** Replicate two and three of peptide counts for proteins identified by IP-mass spec of wild-type, SIMR-1::mCherry::2xHA, and HRDE-2::2xTy1::GFP strains. Anti-HA conjugated beads were used for SIMR-1 IP and wild-type IP, and anti-GFP conjugated beads were used for HRDE-2 IP and wild-type IP. The IPs with anti-HA and anti-GFP beads from wild-type samples had the same results (0 peptide count for the SIMR-1, HRDE-2, HRDE-1, and NRDE-3 proteins). Peptide counts for replicate 1 can be found in Fig. 2A. Full protein lists are in Supplementary Data 1.

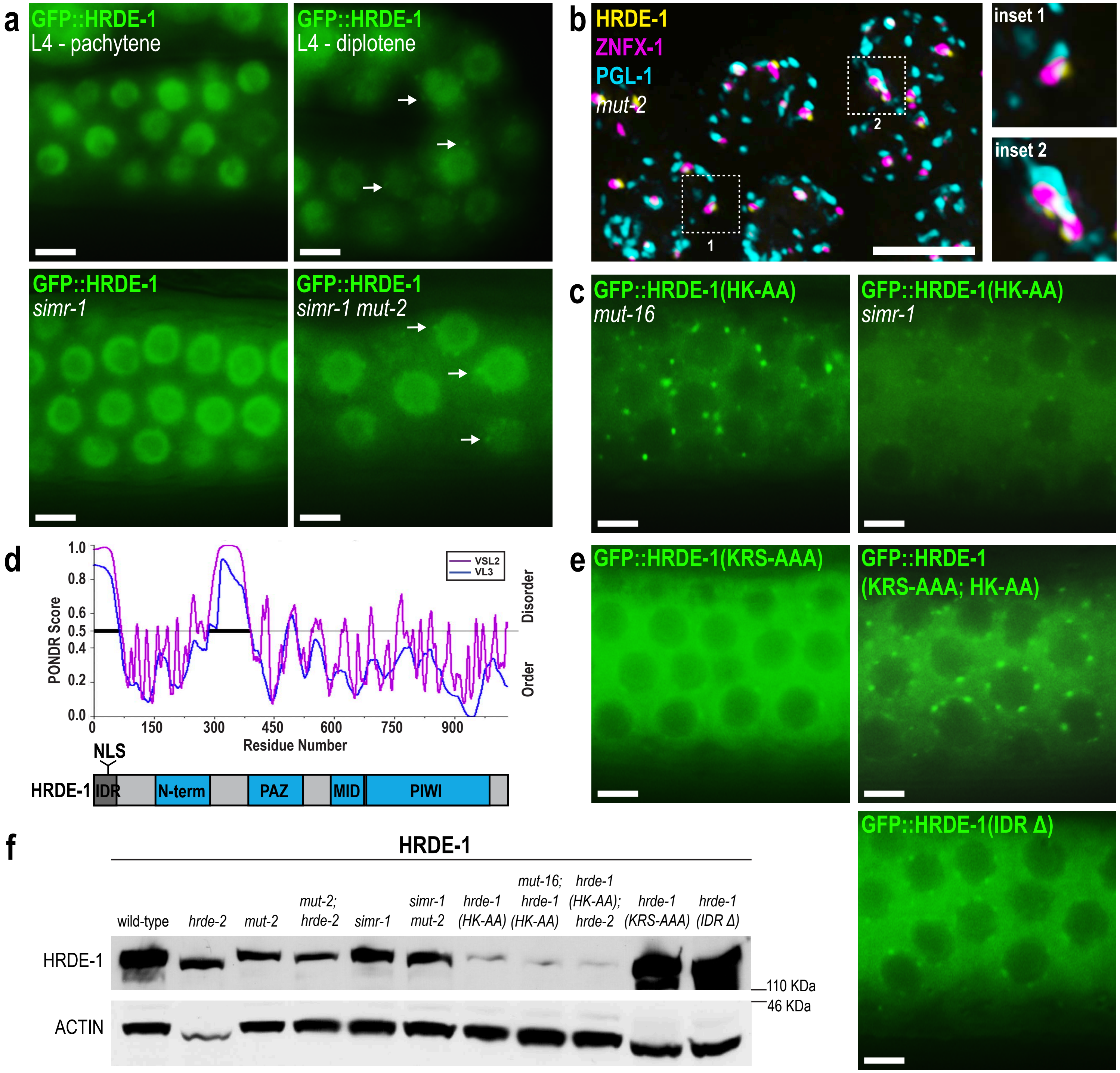

Supplementary Fig. 3 Unloaded HRDE-1 granule localization and expression level in mutants. a Live imaging of GFP::3xFLAG::HRDE-1 in (top, left to right) L4 pachytene, L4 diplotene, (bottom, left to right) *simr-1* mutant, and *simr-1 mut-2* double mutant. Arrow shows that HRDE-1 granule localization in diplotene, and *simr-1 mut-2*. Panels were individually adjusted for brightness and contrast to optimize visualization of germ granules or lack thereof. At least five individual animals were imaged for each strain. Scale bars, 5 µM. **b** Immunofluorescence imaging of GFP::3xFLAG::HRDE-1 and HA::tagRFP::ZNFX-1 in a dissected day-one adult germline from a *mut-2* mutant, using antibodies against FLAG, HA, and PGL-1. All images are projections of 3D projections following deconvolution. At least eight individual germlines were imaged. Scale bars, 5 µM. **c** Live imaging of GFP::3xFLAG::HRDE-1 in *hrde-1(HK-AA); mut-16* double mutants and *hrde-1(HK-AA); simr-1* double mutants. Panels were individually adjusted for brightness and contrast to optimize visualization of germ granules or lack thereof. At least five individual animals were imaged for each strain. Scale bars, 5 µM. **d** Graph displaying disorder tendency for the HRDE-1 protein sequence. The disorder prediction was made using PONDR (http://www.pondr.com) with VSL2 and VSL3 parameters. Regions with PONDR scores of greater than 0.5 indicate disorder and regions with PONDR score less than 0.5 indicate order. The bold line from a.a. 1 to 57 indicates the predicted disordered regions of HRDE-1. Below, a domain diagram for HRDE-1 shows that it contains an N-terminal IDR and NLS and the conserved N-terminal, PAZ, MID, and PIWI domains. **e** Live imaging of GFP::3xFLAG::HRDE-1 in (top, left to right) *hrde-1(KRS-AAA)* NLS mutant, *hrde-1(KRS-AAA; HK-AA)* NLS and small RNA binding double mutant, and (bottom) *hrde-1(IDRΔ)* deletion mutant strains. Panels were individually adjusted for brightness and contrast to optimize visualization of germ granules or lack thereof. At least five individual animals were imaged for each strain. Scale bars, 5 µM. **f** Western blot of HRDE-1 protein level in wild-type, *hrde-2* mutant, *mut-2* mutant, *mut-2; hrde-2* double mutant, *simr-1* mutant, *simr-1 mut-2* double mutant, *hrde-1(HK-AA)* mutant, *mut-16; hrde-1(HK-AA)* double mutant, *hrde-1(HK-AA); hrde-2* double mutant, *hrde-1(KRS-AAA)* NLS mutant, and *hrde-1(IDRΔ)* deletion mutant from day-one adults. Anti-FLAG and anti-Actin antibodies were used for HRDE-1 and Actin respectively.

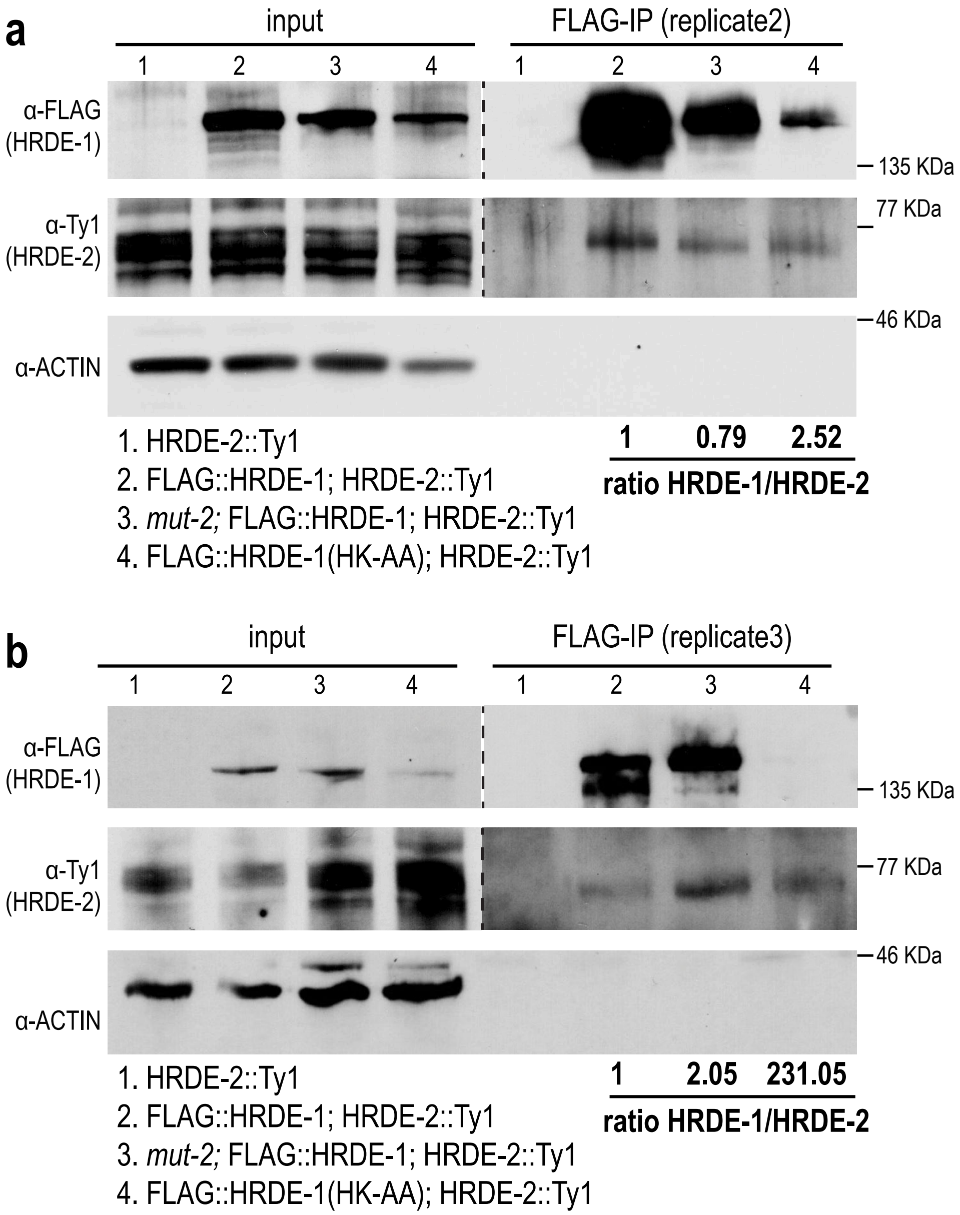

c

HRDE-2 peptide counts from FLAG IP mass-spec

genotype \ replicate #	1	2	3
**Wild-type**	0	0	0
**FLAG::HRDE-1**	NA	1	0
***mut-2;* FLAG::HRDE-1**	0	0	3
**FLAG::HRDE-1(HK-AA)**	3	7	1

Supplementary Fig. 4 HRDE-2 interacts more strongly with unloaded HRDE-1. a-b Two additional replicates of a western blot following immunoprecipitation of HRDE-1 in wild-type, *mut-2* mutants, and *hrde-1(HK-AA)* mutants, where HRDE-1 was tagged with GFP::3xFLAG and HRDE-2 was tagged with 2xTy1::GFP. The HRDE-2::2xTy1::GFP strain was used as a negative control. Anti-FLAG conjugated beads were used to immunoprecipitate GFP::3xFLAG::HRDE-1 and anti-FLAG, Anti-Ty1, and anti-Actin antibodies were used to detect HRDE-1, HRDE-2, and Actin. Band intensities were quantified using ImageJ and normalized to wild-type (lane 2). **c** Peptide counts for the HRDE-2 protein identified by IP-mass spec of wild-type, GFP::3xFLAG::HRDE-1, GFP::3xFLAG::HRDE-1 in the *mut-2* mutant, and GFP::3xFLAG::HRDE-1(HK-AA) in triplicate. Data for GFP::3xFLAG::HRDE-1 replicate 1 is unavailable due to a failure of the mass spec run for this sample. Anti-FLAG conjugated beads were used for IP. Full protein lists are in Supplementary Data 1.

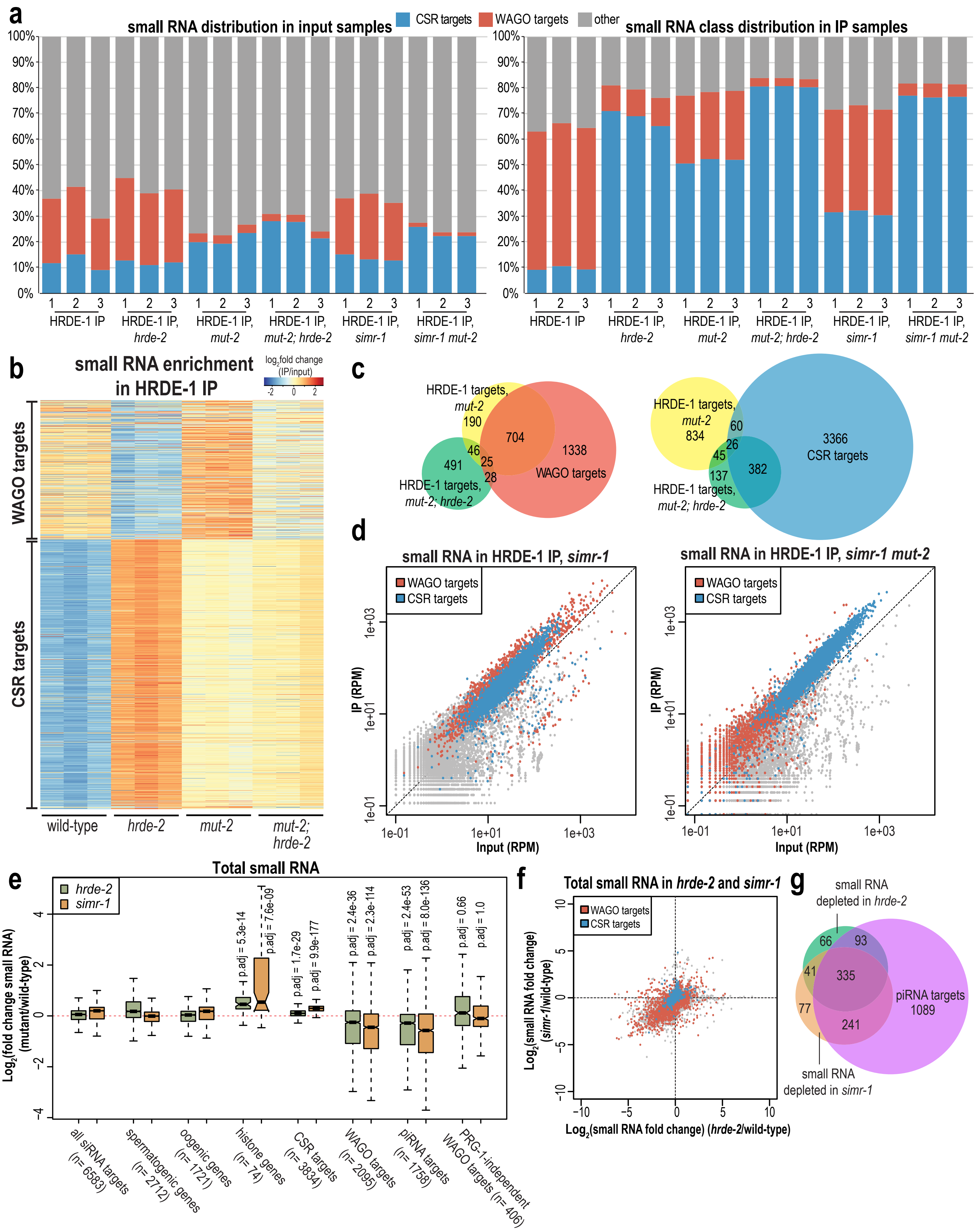

Supplementary Fig. 5 *simr-1* and *hrde-2* mutants alter HRDE-1 target genes. (legend continued on next page)

Supplementary Fig. 5 *simr-1* and *hrde-2* mutants alter HRDE-1 target genes. a Percentage of reads from HRDE-1 input and IP small RNA libraries mapping to CSR-target genes, WAGO-target genes, or other genes in wild-type and the indicated mutants. Each of three replicate libraries are shown separately. **b** Heatmap shows log2(fold change) for small RNAs mapping to WAGO-target and CSR-target genes in HRDE-1 IP compared to input for wild-type, *hrde-2*, *mut-2*, and *mut-2; hrde-2* mutants. All three replicates are shown. **c** Venn diagrams indicate overlap of genes enriched for small RNAs in HRDE-1 IP from *mut-2* and *mut-2; hrde-2* mutants compared to WAGO targets (left) and CSR targets (right). HRDE-1 target genes in *mut-2* and *mut-2; hrde-2* mutants are defined as at least twofold enriched in the IP, with at least 10 RPM in IP samples and a DESeq2 adjusted p value ≤0.05. **d** Normalized HRDE-1 IP compared to input small RNA reads for *simr-1* and *mut-2 simr-1* mutants from day 1 adults. WAGO class and CSR class genes are indicated in red and blue, respectively. One representative replicate is shown. **e** Box plots depicting log2(fold change small RNA abundance) in *hrde-2* and *simr-1* mutants compared to wild-type for three biological replicates. Bolded midline indicates median value, box indicates the first and third quartiles, and whiskers represent the most extreme data points within 1.5 times the interquartile range, excluding outliers. Two-tailed t-tests were performed to determine statistical significance and p values were adjusted for multiple comparisons. Adjusted p values are a comparison of the indicated gene list to all siRNA target genes for each mutant. **f** Scatter plots displaying each gene as a function of its log2 fold change for the level of small RNAs immunoprecipitated by HRDE-1 in *hrde-2* mutant (x-axis) and in *simr-1* mutant (y-axis) compared to wild-type. WAGO-target and CSR-target genes are indicated in red and blue, respectively. **g** Venn diagram indicates overlap of genes depleted of small RNAs in *simr-1* and *hrde-2* mutants compared to genes depleted of small RNAs in a *prg-1* mutant (piRNA target genes). The gene lists are defined as genes that are at least twofold depleted of small RNAs in the mutant compared to wild-type, with at least 10 RPM in wild-type and a DESeq2 adjusted p value ≤0.05.

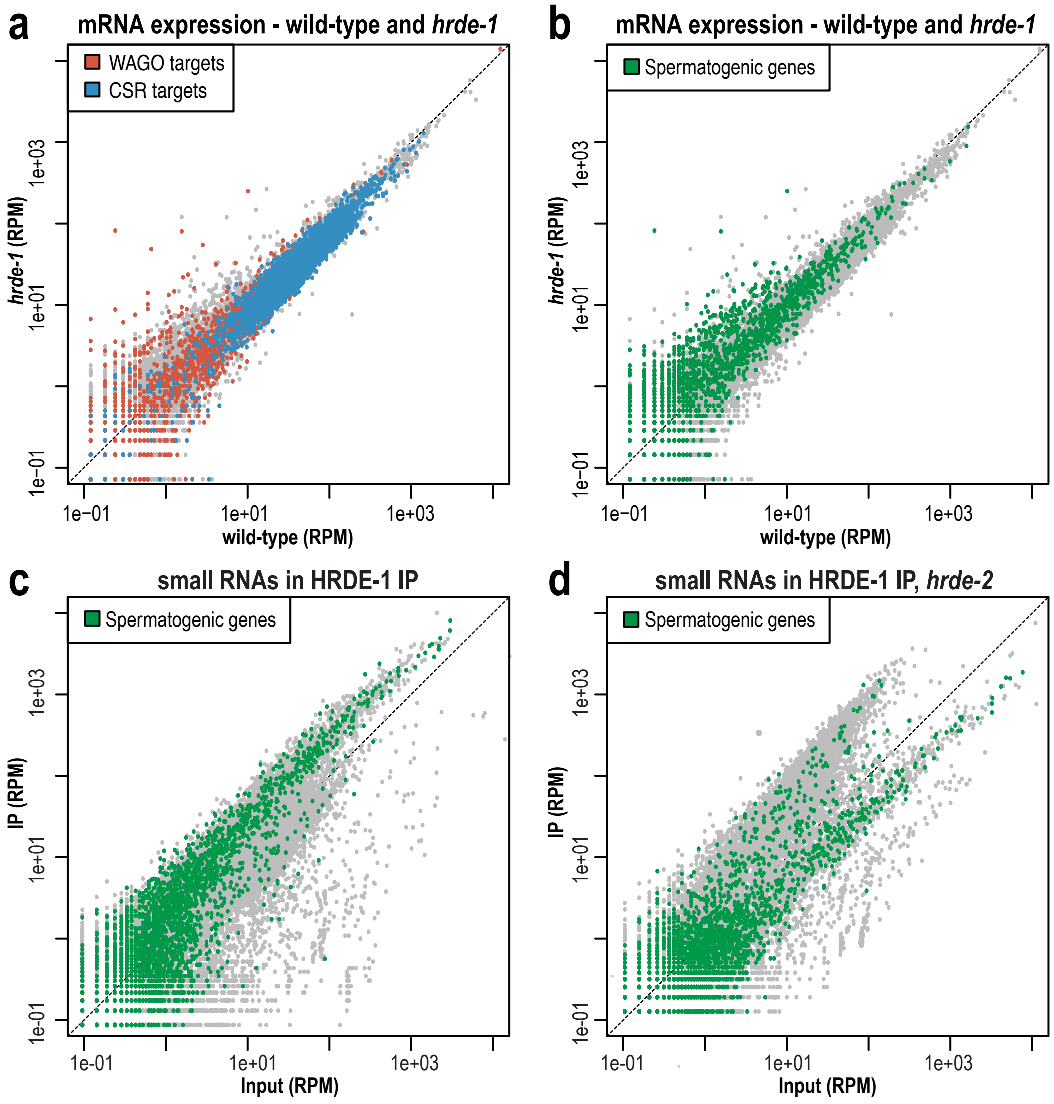

Supplementary Fig. 6 mRNA and small RNA expression in *hrde-1* and *hrde-2* mutants. a Normalized mRNA reads in *hrde-1* mutants compared to wild-type from day 1 adults. WAGO-target and CSR-target genes are indicated in red and blue, respectively. One representative replicate is shown. **b** Normalized mRNA reads in *hrde-1* mutants compared to wild-type from day 1 adults. Spermatogenic genes are indicated in green. One representative replicate is shown. **c** Normalized HRDE-1 IP compared to input small RNA reads in wild-type animals from day 1 adults. Spermatogenic genes are indicated in green. One representative replicate is shown. **d** Normalized HRDE-1 IP compared to input small RNA reads in *hrde-2* mutant animals from day 1 adults. Spermatogenic genes are indicated in green. One representative replicate is shown.

a

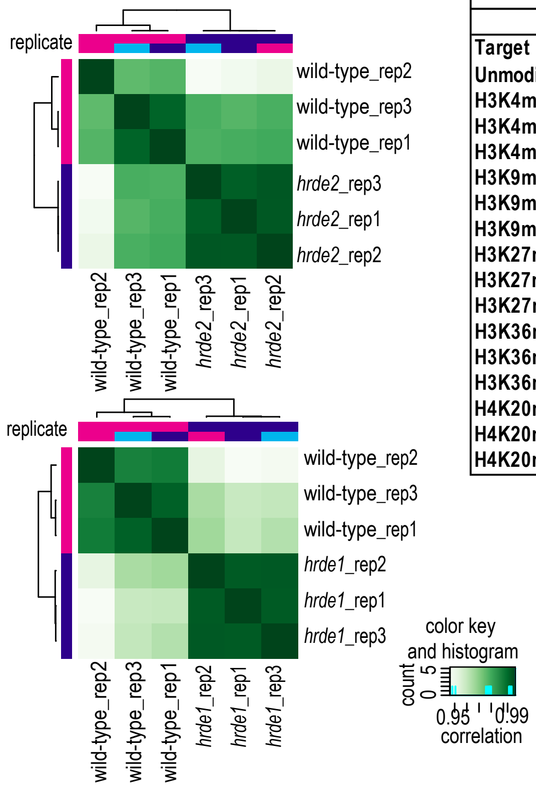

b

H3K9me3 antibody specificity table

	wild-type				*hrde1*				*hrde2*			
**Target**	**IgG**	**rep1**	**rep2**	**rep3**	**IgG**	**rep1**	**rep2**	**rep3**	**IgG**	**rep1**	**rep2**	**rep3**
**Unmodified**	5.7	3.1	3.3	9.0	4.2	3.2	3.2	3.0	4.9	3.2	3.4	3.2
**H3K4me1**	8.5	3.6	5.2	14.2	5.6	4.2	4.4	3.8	5.2	3.8	4.3	3.9
**H3K4me2**	6.3	2.9	3.1	10.4	3.7	3.8	3.5	2.6	7.0	3.2	3.2	2.9
**H3K4me3**	6.4	21.0	28.7	41.6	5.9	30.8	27.7	24.5	7.8	29.5	33.3	27.6
**H3K9me1**	2.9	1.5	1.2	8.8	2.0	1.5	1.5	1.5	0.9	1.6	1.6	1.5
**H3K9me2**	4.8	3.4	4.2	9.2	2.2	4.4	3.5	3.5	1.3	4.6	4.4	4.3
**H3K9me3**	4.4	100.0	100.0	100.0	8.8	100.0	100.0	100.0	8.9	100.0	100.0	100.0
**H3K27me1**	4.5	2.4	2.5	8.7	3.7	2.7	2.5	2.0	2.7	2.3	2.2	2.6
**H3K27me2**	4.8	7.9	10.1	23.1	3.8	10.6	6.7	7.8	6.0	10.0	8.2	7.5
**H3K27me3**	7.0	96.5	108.2	106.5	10.0	109.5	107.4	105.8	11.0	112.4	113.7	105.5
**H3K36me1**	8.4	4.2	5.3	9.5	4.4	4.5	4.9	4.3	4.9	4.5	4.1	4.1
**H3K36me2**	8.7	4.3	4.0	10.1	11.3	4.6	4.7	4.2	4.6	4.8	4.6	5.2
**H3K36me3**	6.5	4.6	5.6	9.2	8.5	7.3	5.6	5.3	9.7	6.2	6.2	5.7
**H4K20me1**	4.9	2.7	3.0	10.6	6.0	2.9	2.9	2.5	4.5	3.2	2.7	2.8
**H4K20me2**	6.9	3.1	4.2	75.5	6.3	3.6	3.3	2.8	9.5	3.1	3.7	3.6
**H4K20me3**	9.1	124.6	151.0	175.7	13.7	180.6	164.2	165.6	11.0	147.4	162.6	140.5

c

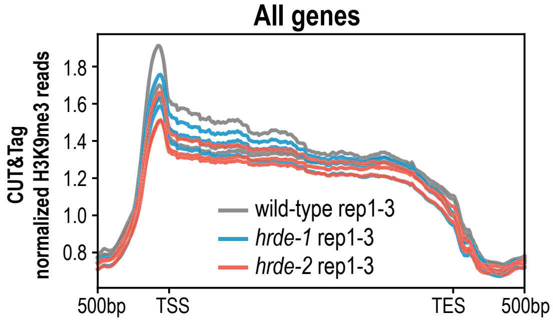

d

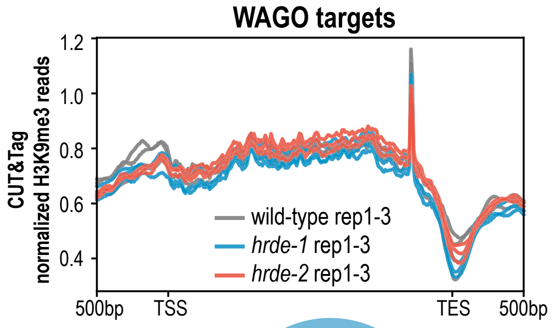

e

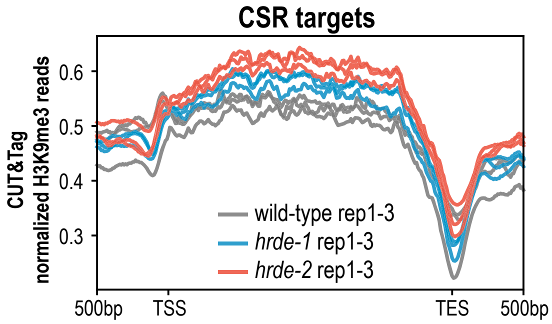

f

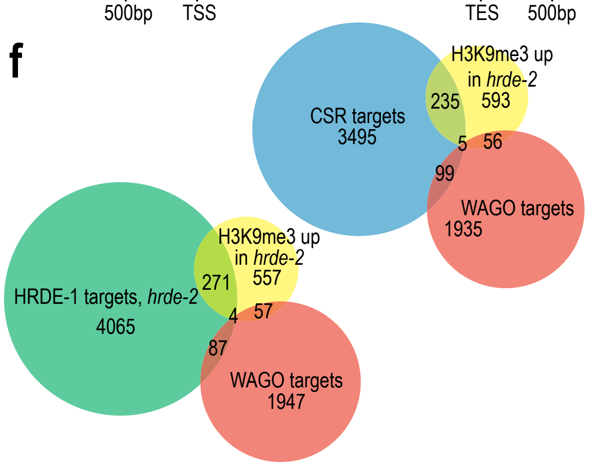

g

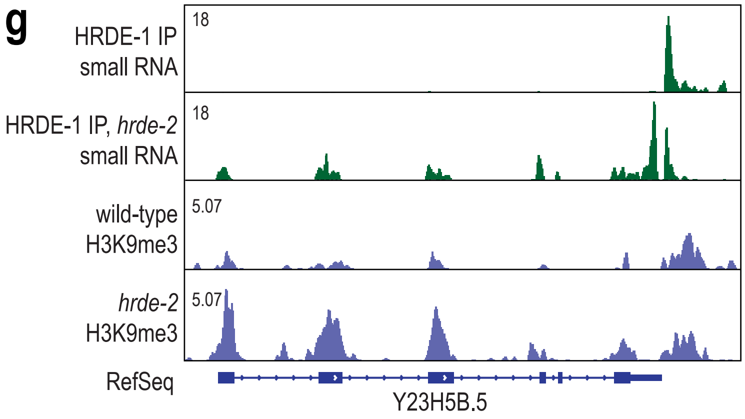

Supplementary Fig. 7 H3K9me3 CUT&Tag data analysis for *hrde-1* and *hrde-2* mutants. a Heatmap showing clustering of the three CUT&Tag replicates. Top: wild-type and *hrde-2* mutants. Bottom: wild-type and *hrde-1* mutants. **b** Anti-H3K9me3 antibody specificity table generated using K-MetStat Panel spike-in. 16 different K-methylation PTM states: mono-, di-, and trimethylation at H3K4, H3K9, H3K27, H3K36, & H4K20, as well as unmodified control alignment rate normalized to H3K9me3 were represented. The H3K9me3 antibody used also shows high binding to two other trimethylated histone tails, H3K27me3 and H4K20me3. **c** Density plot of H3K9me3 level on all *C. elegans* genes in wild-type (grey), *hrde-1* mutants (blue), and *hrde-2* mutants (red). 500bp upstream transcription start site (TSS) and downstream transcription end site (TES) were plotted using normalized H3K9me3 reads. Each of three replicates are represented as individual lines. **d** Density plot of H3K9me3 level on WAGO-target genes in wild-type (grey), *hrde-1* mutants (blue), and *hrde-2* mutants (red). 500bp upstream transcription start site (TSS) and downstream transcription end site (TES) were plotted using normalized H3K9me3 reads. Each of three replicates are represented as individual lines. **e** Density plot of H3K9me3 level on CSR-target genes in wild-type (grey), *hrde-1* mutants (blue), and *hrde-2* mutants (red). 500bp upstream transcription start site (TSS) and downstream transcription end site (TES) were plotted using normalized H3K9me3 reads. Each of three replicates are represented as individual lines. **f** Venn diagram showing overlap of genes with increased H3K9me3 level in *hrde-2* mutant compared to WAGO-target genes, CSR-target genes or genes targeted by HRDE-1-bound small RNAs in a *hrde-2* mutant. **g** Normalized small RNA and H3K9me3 read distribution across a CSR target gene (Y23H5B.5) in wild-type and *hrde-2* mutants. One representative replicate shown.

a

genotype	HRDE-1 localization	HRDE-1 loading	Class of HRDE-1-bound small RNA
wild-type	nucleus	yes	WAGO
hrde-2	nucleus	yes	CSR
mut-2	nucleus and granule	partially	WAGO and CSR
mut-2; hrde-2	nucleus	yes	CSR
hrde-1(HK-AA)	granule	no	N/A
hrde-1(HK-AA); hrde-2	cytosol	no	N/A

b ***hrde-2* mutant**

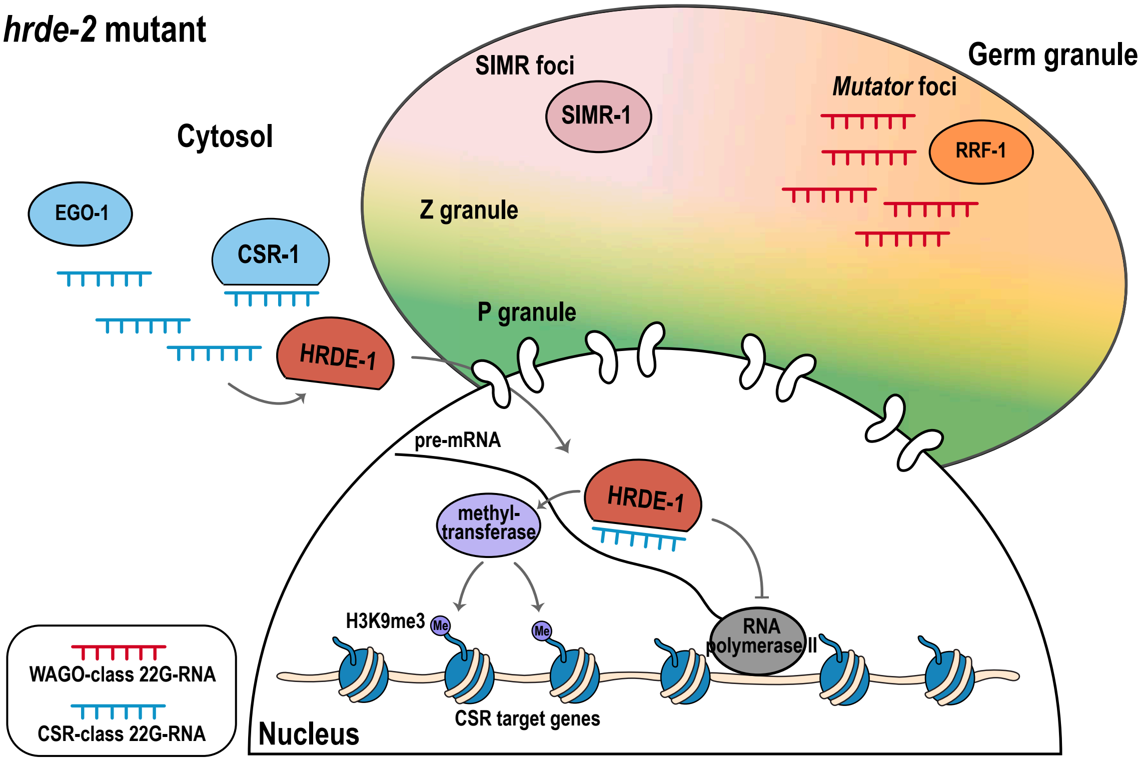

Supplementary Fig. 8 HRDE-2 promotes germ granule localization and small RNA binding for HRDE-1. a A table summarizes the HRDE-1 localization and small RNA loading pattern in the wild-type, *hrde-2* mutant, *mut-2* mutant, *mut-2; hrde-2* mutant, *hrde-1(HK-AA)* mutant, and *hrde-1(HK-AA); hrde-2* mutant. **b** A model of HRDE-1 loading in the *hrde-2* mutant: because unloaded HRDE-1 is not retained in germ granules, it encounters and loads CSR-class 22G-RNAs in the cytosol, allowing it to translocate into the nucleus and target CSR-target genes for transcriptional silencing.

Supplementary Fig. 1f-top: HRDE-2 protein levels in wild-type and mutants

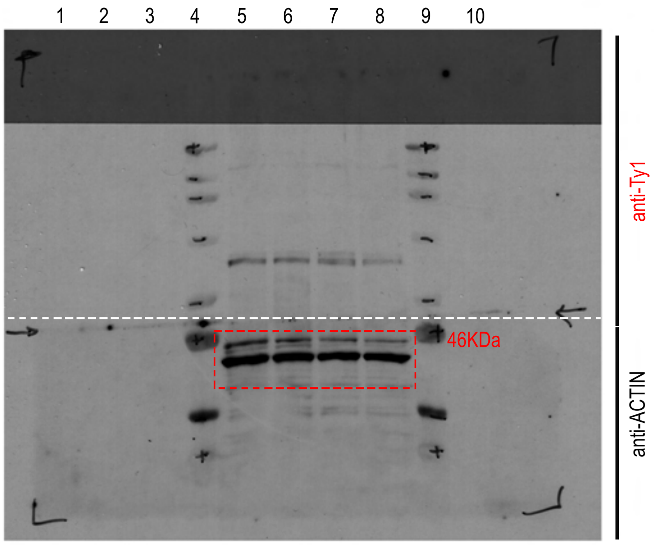

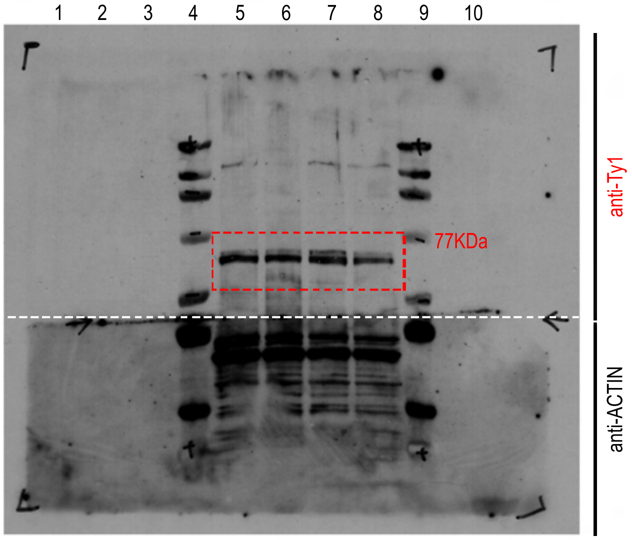

Supplementary Fig. 1f-top lanes

1. empty
2. empty
3. empty
4. ladder
5. HRDE-2::Ty1
6. HRDE-2:Ty1, SIMR-1::HA
7. HRDE-2::Ty1, *simr-1*
8. HRDE-2::Ty1, *mut-16*
9. ladder
10. empty

Supplementaty Fig. 1f-bottom: SIMR-1 protein levels in wild-type and *hrde-2* mutant

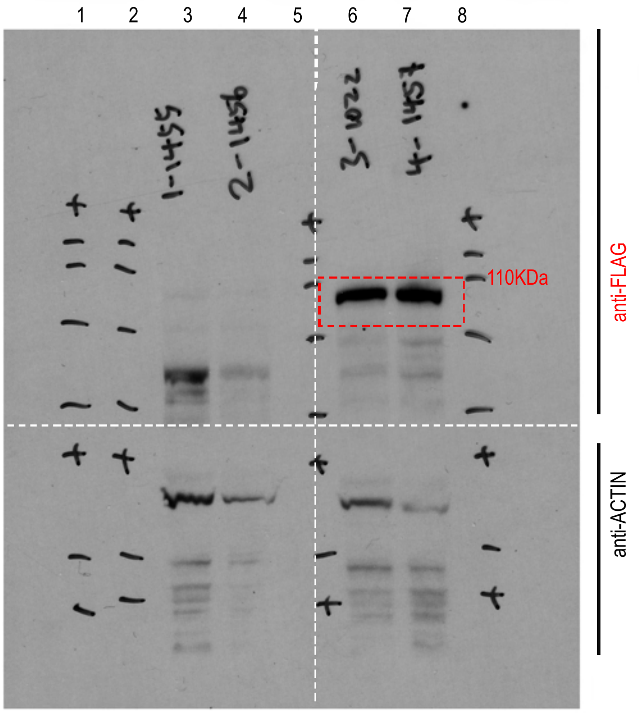

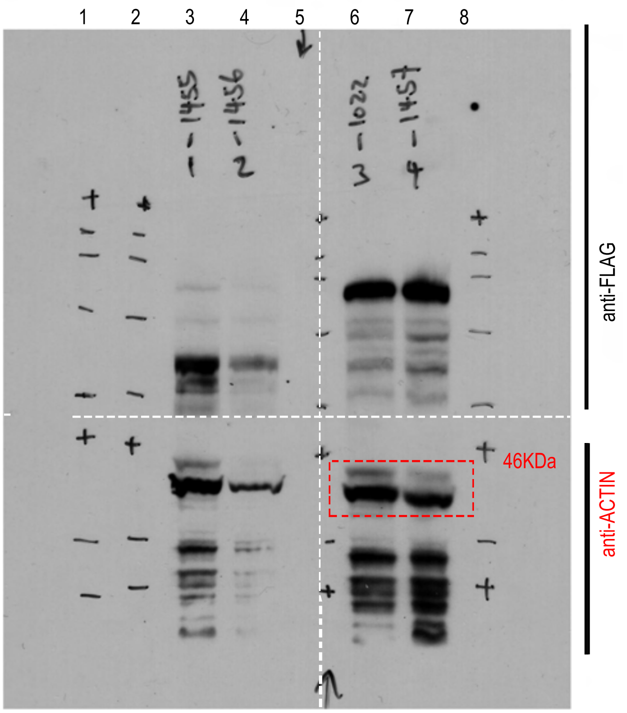

Supplementary Fig. 1f-bottom lanes

1. ladder
2. ladder
3. HRDE-2::Ty1, SIMR-1::HA
4. HRDE-2::Ty1, *simr-1*
5. ladder
6. SIMR-1::GFP
7. SIMR-1::GFP, *hrde-2*
8. ladder

Supplementary Fig. 2a: HRDE-2 and SIMR-1 co-IP -1

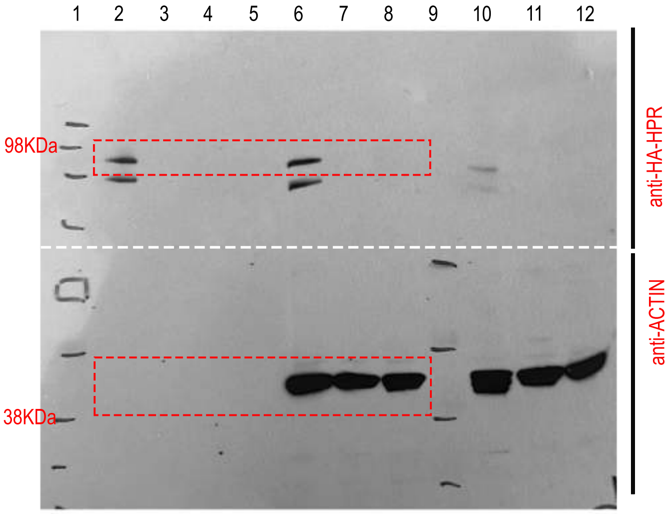

(vertically flipped in Supplementary Fig. 2a)

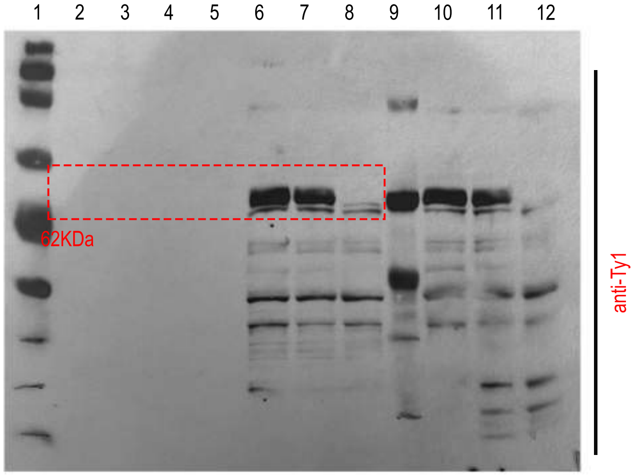

(vertically flipped in Supplementary Fig. 2a)

Supplementaty Fig. 2b: HRDE-2 and SIMR-1 co-IP -2

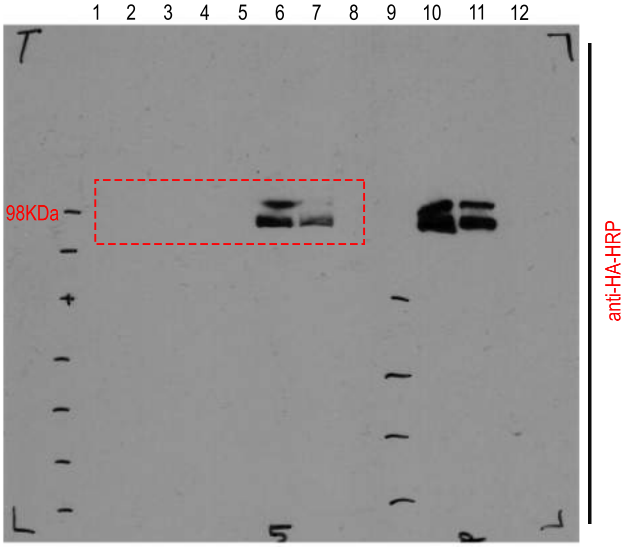

(vertically flipped in Supplementary Fig. 2b)

Supplementary Fig. 2a lanes

1. ladder
2. HRDE-2::Ty1, SIMR-1::HA. IP
3. HRDE-2::Ty1. IP
4. wild-type. IP
5. empty
6. HRDE-2::Ty1, SIMR-1::HA. Input
7. HRDE-2::Ty1. Input
8. wild-type. Input
9. ladder
10. HRDE-2::Ty1, SIMR-1::HA. Lysate
11. HRDE-2::Ty1. Lysate
12. wild-type. Lysate

Supplementary Fig. 2b lanes

1. ladder
2. HRDE-2::Ty1, SIMR-1::HA. IP
3. SIMR-1::HA. IP
4. wild-type. IP
5. empty
6. HRDE-2::Ty1, SIMR-1::HA. Input
7. SIMR-1::HA. Input
8. wild-type. Input
9. ladder
10. HRDE-2::Ty1, SIMR-1::HA. Lysate
11. SIMR-1::HA. Lysate
12. wild-type. Lysate

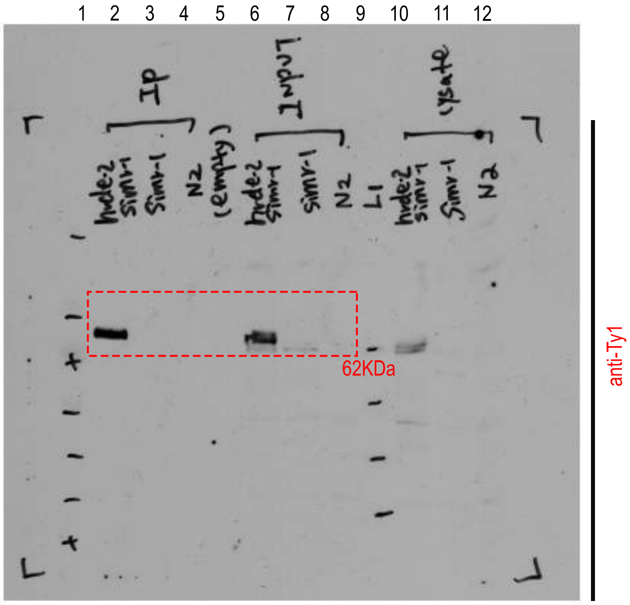

(vertically flipped in Supplementary Fig. 2b)

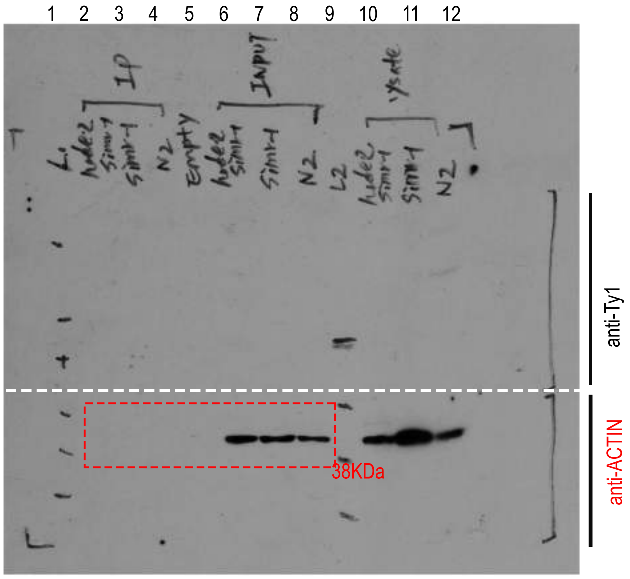

(vertically flipped in Supplementary Fig. 2b)

Supplementary Fig. 3d: HRDE-1 protein levels in wild-type and different mutants

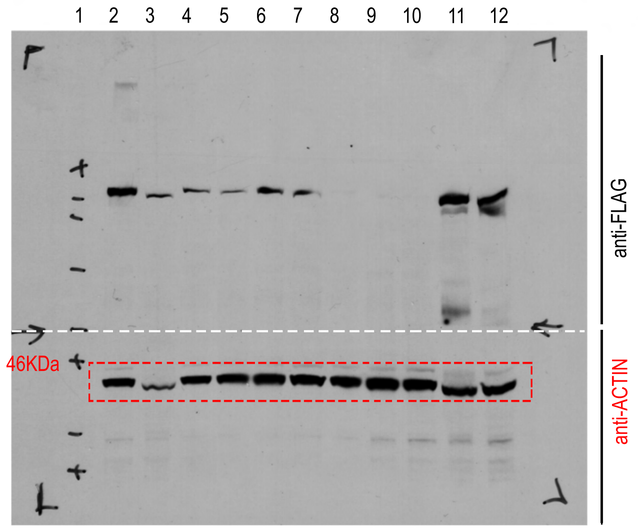

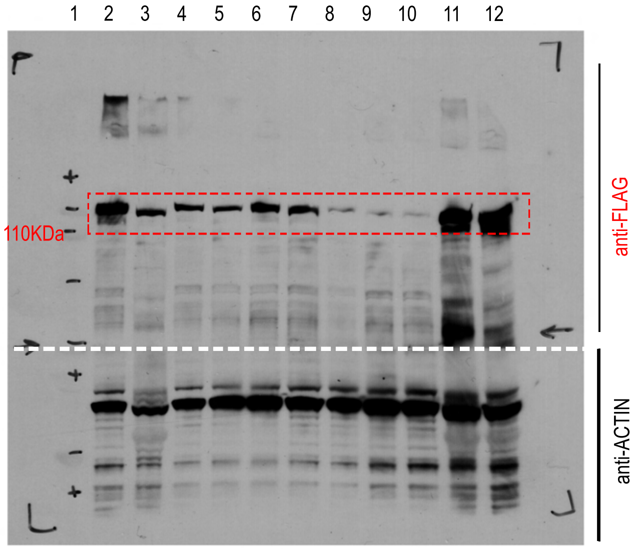

Supplementary Figure. 3d lanes

1. ladder
2. FLAG::HRDE-1
3. FLAG::HRDE-1, *hrde-2*
4. FLAG::HRDE-1, *mut-2*
5. FLAG::HRDE-1, *mut-2; hrde-2*
6. FLAG::HRDE-1, *simr-1*
7. FLAG::HRDE-1, *simr-1 mut-2*
8. FLAG::HRDE-1(HK-AA)
9. FLAG::HRDE-1(HK-AA), *mut-16*
10. FLAG::HRDE-1(HK-AA), *hrde-2*
11. FLAG::HRDE-1(KRS-AAA)
12. FLAG::HRDE-1(IDR deletion)

Supplementary Fig. 4a: HRDE-1, HRDE-2 co-IP in mutants, replicate 2

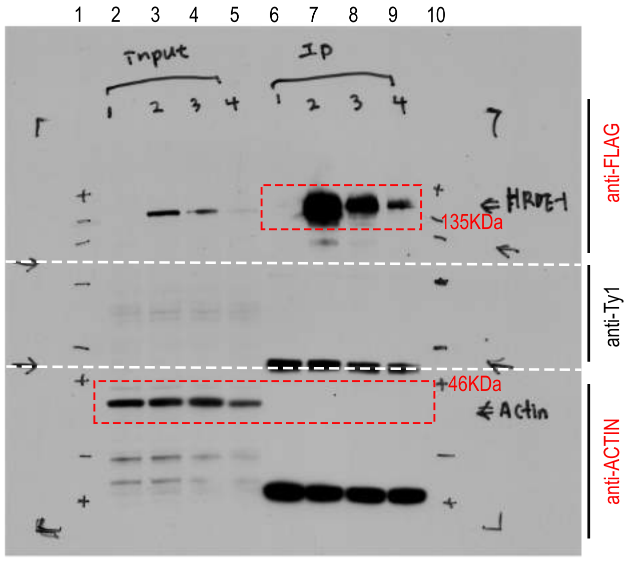

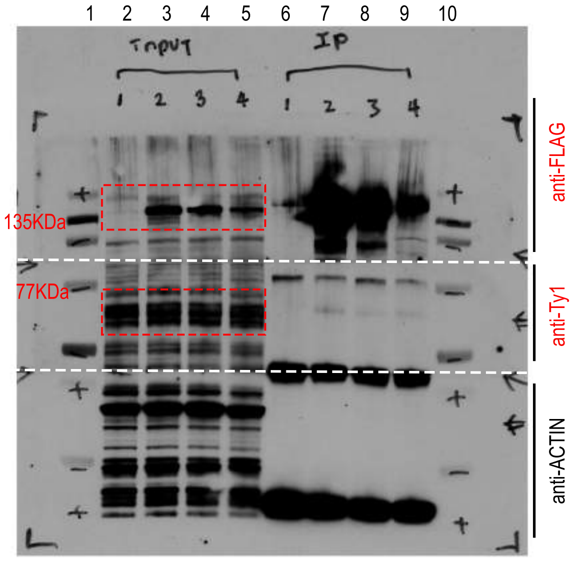

Supplementary Fig. 4a and Fig. 4b lanes
1. Ladder
2. HRDE-2::Ty1. Input
3. HRDE-2::Ty1; FLAG::HRDE-1. Input
4. HRDE-2::Ty1; FLAG::HRDE-1; mut-2. Input
5. HRDE-2::Ty1; FLAG::HRDE-1(HK-AA). Input
6. HRDE-2::Ty1..IP
7. HRDE-2::Ty1; FLAG::HRDE-1. IP
8. HRDE-2::Ty1; FLAG::HRDE-1; mut-2. IP
9. HRDE-2::Ty1; FLAG::HRDE-1(HK-AA). IP
10. Ladder

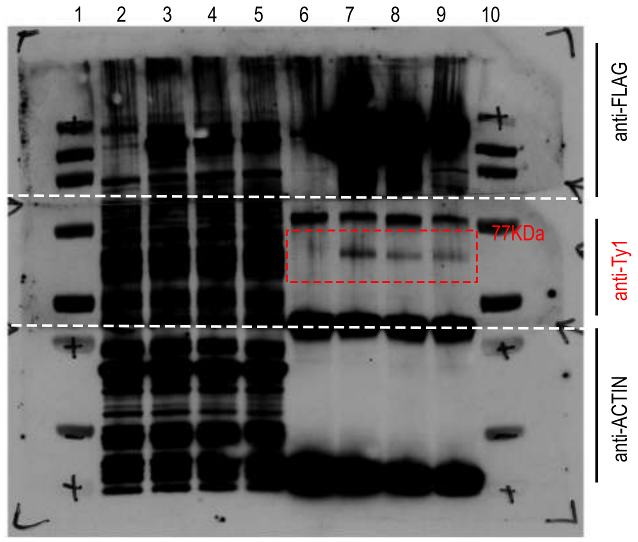

Supplementary Fig. 4b: HRDE-1, HRDE-2 co-IP in mutants, replicate 3

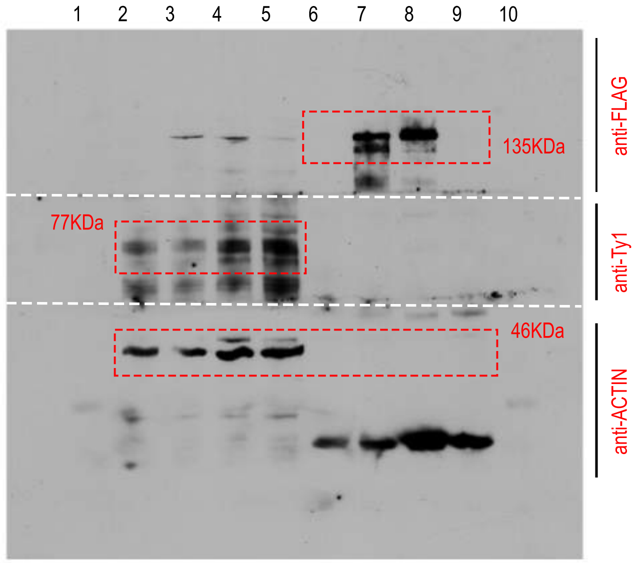

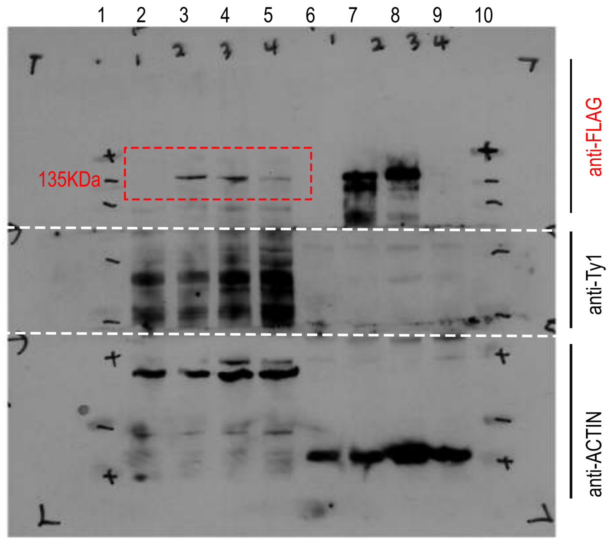

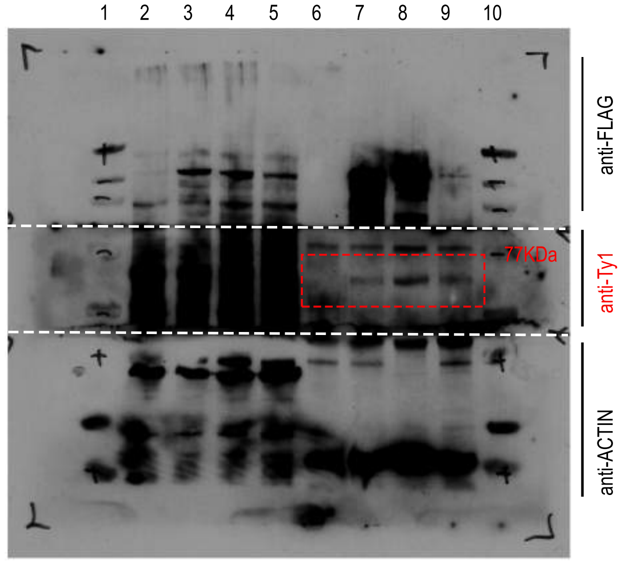

Supplement: Supplementary file 1 — Supplementary Information [file 41467_2024_45245_MOESM1_ESM.pdf]
