# Supplementary material for: HRDE-2 drives small RNA specificity for the nuclear Argonaute protein HRDE-1
Source: Nat Commun. 2024 Feb 1;15:957. doi: 10.1038/s41467-024-45245-8 (PMC10834429; doi:10.1038/s41467-024-45245-8)
Supplement: Supplementary file 3 — Description of Additional Supplementary Files [file 41467_2024_45245_MOESM3_ESM.pdf]

Description of Additional Supplementary Files

File Name: Supplementary Data 1
Description: Protein interactions identified in this study.

File Name: Supplementary Data 2
Description: Reagents, resources, and strains used in this study.

File Name: Supplementary Data 3
Description: Oligonucleotides sequences used in this study.

File Name: Supplementary Data 4
Description: Small RNA enrichment in HRDE-1 immunoprecipitations.

File Name: Supplementary Data 5
Description: Sequencing library statistics.

File Name: Supplementary Data 6
Description: mRNA expression in *hrde-1(tm1200)* and *hrde-2(qe20)* mutant animals.

File Name: Supplementary Data 7
Description: H3K9me3 level in *hrde-1(tm1200)* and *hrde-2(qe20)* mutant animals.
