# Supplementary material for: HRDE-2 drives small RNA specificity for the nuclear Argonaute protein HRDE-1
Source: Nat Commun. 2024 Feb 1;15:957. doi: 10.1038/s41467-024-45245-8 (PMC10834429; doi:10.1038/s41467-024-45245-8)
Supplement: Supplementary file 11 — Reporting Summary [file 41467_2024_45245_MOESM11_ESM.pdf]

nature portfolio

Corresponding author(s): Carolyn M. Phillips

Last updated by author(s): Jan 1, 2024

Reporting Summary

Nature Portfolio wishes to improve the reproducibility of the work that we publish. This form provides structure for consistency and transparency in reporting. For further information on Nature Portfolio policies, see our Editorial Policies and the Editorial Policy Checklist.

Statistics

For all statistical analyses, confirm that the following items are present in the figure legend, table legend, main text, or Methods section.

n/a	Confirmed	
☐	☒	The exact sample size (*n*) for each experimental group/condition, given as a discrete number and unit of measurement
☐	☒	A statement on whether measurements were taken from distinct samples or whether the same sample was measured repeatedly
☐	☒	The statistical test(s) used AND whether they are one- or two-sided<br>*Only common tests should be described solely by name; describe more complex techniques in the Methods section.*
☐	☒	A description of all covariates tested
☐	☒	A description of any assumptions or corrections, such as tests of normality and adjustment for multiple comparisons
☐	☒	A full description of the statistical parameters including central tendency (e.g. means) or other basic estimates (e.g. regression coefficient) AND variation (e.g. standard deviation) or associated estimates of uncertainty (e.g. confidence intervals)
☐	☒	For null hypothesis testing, the test statistic (e.g. *F*, *t*, *r*) with confidence intervals, effect sizes, degrees of freedom and *P* value noted<br>*Give P values as exact values whenever suitable.*
☒	☐	For Bayesian analysis, information on the choice of priors and Markov chain Monte Carlo settings
☒	☐	For hierarchical and complex designs, identification of the appropriate level for tests and full reporting of outcomes
☒	☐	Estimates of effect sizes (e.g. Cohen's *d*, Pearson's *r*), indicating how they were calculated

Our web collection on statistics for biologists contains articles on many of the points above.

Software and code

Policy information about availability of computer code

Data collection	No software was used.
Data analysis	For small RNA libraries, sequences were parsed from adapters and quality filtered using FASTX-Toolkit (version 0.0.13) (Hannon, G.J. (2010) FASTX-Toolkit. http://hannonlab.cshl.edu/fastx_toolkit). Contamination from reads mapping to 18-mer and 28-mer size standards were filtered out using Cutadapt (version 3.4) (Martin 2011). Filtered reads were mapped to the C. elegans genome, WS258, using Bowtie2 (version 2.5.0) (Langmead and Salzberg 2012). Mapped reads were assigned to genomic features using featureCounts which is part of the Subread package (version 2.0.1) (Liao et al. 2014). Differential expression analysis was performed using Deseq2 (version 1.38.3) (Love et al. 2014). Deeptools (version 3.5.1) was used to generate metagene plots with BPM (per bin) normalization parameter (Bins Per Million = number of reads per bin/sum of all reads per bin in millions). To define gene lists from IP experiments, a twofold-change cutoff, a DESeq2 adjusted p value of ≤0.05, and at least 10 RPM in the IP libraries were required to identify genes with significant changes in small RNA levels. The all siRNA targets list (6582 targets) in Supplementary Fig. 5E was defined by genes having at least 10RPM in both hrde-2 and simr-1 mutants.<br>For mRNA libraries, adapter sequences were trimmed using Trimmomatic (version 0.39) (Bolger et al. 2014) and mapped to the C. elegans genome, WS258, using HISAT2 (version 2.2.1) (Kim et al. 2019). Mapped reads for genomic features were counted using featureCounts which is part of the Subread package (version 2.0.1) (Liao et al. 2014). Differential expression analysis was performed using Deseq2 (version 1.38.3) (Love et al. 2014). The all genes list (19782 genes) in Fig. 6C was defined by genes having more than 0 RPM in both hrde-1 and hrde-2 mutants.<br>For CUT&Tag libraries, reads were mapped to the C. elegans genome, WS258, using bowtie2 (version 2.5.0) (Langmead and Salzberg 2012). Mapped reads for genomic features were counted using featureCounts which is part of the Subread package (version 2.0.1) (Liao et al. 2014). Bedtools (version 2.30.0) was used to generate the density plots (Quinlan and Hall 2010). IGV (version 2.12.0) was used to visualize the H3K9me3 and small RNA level(Robinson et al. 2011). SEACR (version 1.3) was used to call peaks (Meers et al. 2019). DiffBind (version 3.17) was used for differential expression analysis (Stark and Brown). Deeptools (version 3.5.1) was used to generate metagene plots with BPM (per

bin) normalization parameter (Bins Per Million = number of reads per bin/sum of all reads per bin in millions). The all genes list (41023 genes) in Fig. 7F was defined by genes having more than 0 RPM in both wild-type and hrde-2 mutants.
Venn diagrams were generated using BioVenn (Hulsen et al. 2008). Sequencing data is summarized in the Supplemental Table 7.

For manuscripts utilizing custom algorithms or software that are central to the research but not yet described in published literature, software must be made available to editors and reviewers. We strongly encourage code deposition in a community repository (e.g. GitHub). See the Nature Portfolio guidelines for submitting code & software for further information.

Data

Policy information about availability of data

All manuscripts must include a data availability statement. This statement should provide the following information, where applicable:

- Accession codes, unique identifiers, or web links for publicly available datasets
- A description of any restrictions on data availability
- For clinical datasets or third party data, please ensure that the statement adheres to our policy

The RNA sequencing and CUT&Tag sequencing data generated in this study are available through Gene Expression Omnibus (GEO) under accession code GSE239291 [https://www.ncbi.nlm.nih.gov/geo/query/acc.cgi?acc=GSE239291]. The mass spectrometry proteomics data generated in this study are available in the MassIVE repository with the dataset identifier MSV000092546[https://massive.ucsd.edu/ProteoSAFe/dataset.jsp?task=15befb26a09d4f678779bbbdcfa39c8a].
CSR-target genes, WAGO-target genes, spermatogenesis-enriched genes, and oogenesis-enriched genes were previously described (Nguyen and Phillips 2021; Manage et al. 2020; Ortiz et al. 2014).
Source data are provided with this paper.

Research involving human participants, their data, or biological material

Policy information about studies with human participants or human data. See also policy information about sex, gender (identity/presentation), and sexual orientation and race, ethnicity and racism.

Reporting on sex and gender	N/A
Reporting on race, ethnicity, or other socially relevant groupings	N/A
Population characteristics	N/A
Recruitment	N/A
Ethics oversight	N/A

Note that full information on the approval of the study protocol must also be provided in the manuscript.

Field-specific reporting

Please select the one below that is the best fit for your research. If you are not sure, read the appropriate sections before making your selection.

☒ Life sciences ☐ Behavioural & social sciences ☐ Ecological, evolutionary & environmental sciences

For a reference copy of the document with all sections, see nature.com/documents/nr-reporting-summary-flat.pdf

Life sciences study design

All studies must disclose on these points even when the disclosure is negative.

Sample size	small RNA, mRNA, and CUT&Tag seq data analysis: 3 biological replicates (see Supplementary Table 5). Sample sizes were not predetermined by statistical methods, but by conventional requirements in the respective fields.
Data exclusions	No data was excluded
Replication	All immunofluorescence and live imaging experiments were performed at least two times, and at least 5 germlines were images per sample, per condition, with similar results. All co-immunoprecipitation, western blots and small RNA binding assay were performed at least two times with similar results. Additional information about number of replicates for specific experiments can be found in the figure legends.
Randomization	For all experiments, control and experimental samples were treated in parallel. Animals were chosen randomly from plates for experiments such as live imaging and immunofluorescence imaging.
Blinding	Blinding was not used in this study. The experiments in this study were not subjective.

Reporting for specific materials, systems and methods

We require information from authors about some types of materials, experimental systems and methods used in many studies. Here, indicate whether each material, system or method listed is relevant to your study. If you are not sure if a list item applies to your research, read the appropriate section before selecting a response.

Materials & experimental systems

n/a	Involved in the study
☐	☒ Antibodies
☒	☐ Eukaryotic cell lines
☒	☐ Palaeontology and archaeology
☐	☒ Animals and other organisms
☒	☐ Clinical data
☒	☐ Dual use research of concern
☒	☐ Plants

Methods

n/a	Involved in the study
☒	☐ ChIP-seq
☒	☐ Flow cytometry
☒	☐ MRI-based neuroimaging

Antibodies

Antibodies used

Anti-FLAG M2 (Mouse monoclonal) Sigma Aldrich Cat# F1804, RRID:AB_262044. Dilution: 1:1000 Western blot.
Anti-HA 3F10 (Rat monoclonal) Roche Cat# 11867423001, RRID:AB_390918. Dilution: 1:500 immunofluorescence imaging.
Anti-HA 3F10 Peroxidase (Rat monoclonal) Roche Cat# 12013819001, RRID:AB_390917. Dilution: 1:1000 Western blot.
Anti-Myc (9E10) (Mouse monoclonal) Thermo Fisher Cat# 13-2500. Dilution: 1:100 immunofluorescence imaging.
Anti-PGL-1 (Mouse monoclonal) DSHB Cat# K76, RRID:AB_531836. Dilution: 1:100 immunofluorescence imaging.
Anti-GFP (Rabbit polyclonal) Thermo Fisher Cat# A-11122, RRID:AB_221569. Dilution: 1:500 immunofluorescence imaging.
Anti-Ty1(BB2) (Mouse monoclonal) Thermo Fisher Cat# MA5-23513. Dilution: 1:2000 Western blot.
Anti-H3K9me3 (Rabbit polyclonal) ChIP Grade Abcam Cat# ab8898. Dilution: 1:100 CUT&Tag.
Anti-H3K27me3 C36B11 (Rabbit monoclonal) Cell signaling Cat# 9733T. Dilution: 1:50 CUT&Tag.
Goat anti-mouse IgG Alexa Fluor 488 Thermo Fisher Cat# A-11029, RRID:AB_138404. Dilution: 1:1000 immunofluorescence imaging.
Goat anti-Rat IgG Alexa Fluor 555 Thermo Fisher Cat# A-21434, RRID:AB_2535855. Dilution: 1:1000 immunofluorescence imaging.
Goat anti-mouse IgM Alexa Fluor 647 Thermo Fisher Cat# A21238, RRID:AB_1500930. Dilution: 1:500 immunofluorescence imaging.
Goat anti-mouse IgG Alexa Fluor 647 Thermo Fisher Cat# A21236. Dilution: 1:500 immunofluorescence imaging.
Guinea Pig anti-Rabbit IgG (Guinea Pig polyclonal) Antibodies-Online Cat# ABIN101961. Dilution: 1:100 CUT&Tag.
Anti-actin IgG (Mouse monoclonal) Abcam Cat# ab3280, RRID:AB_303668. Dilution: 1:10000 Western blot.
Goat anti-mouse IgG Secondary HRP Thermo Fisher Cat# A16078, RRID:AB_2534751. Dilution: 1:10000 Western blot.
Goat anti-rabbit Secondary HRP Thermo Fisher Cat# A16110, RRID: AB_2534782. Dilution: 1:10000 Western blot.
Anti-FLAG M2 Affinity Matrix Sigma Aldrich Cat# A2220, RRID:AB_10063035. No dilution immunoprecipitation.
Anti-HA 3F10 Affinity Matrix Sigma Aldrich Cat# 11815016001, RRID:AB_390914. No dilution immunoprecipitation.
Anti-GFP Agarose (RQ2) MBL Cat# D153-8. No dilution immunoprecipitation.

Validation

All of the commercial purchased antibodies were validated in previous studies and by the manufacturer, more specifically:
Anti-FLAG M2 was validated by western blot detecting for the N-term FLAG-BAP fusion protein in either bacteria, mammalian, or plant extract, then directly detected using anti-mouse IgG peroxidase, and visualized using HRP chemiluminescent substrates (chrome-extension://efaidnbmnnnibpcajpcglclefindmkaj/https://www.sigmaaldrich.com/deepweb/assets/sigmaaldrich/product/documents/754/849/anti-flag-2poster.pdf).
Anti-HA 3F10 was validated by western blot detecting for purified HA-tagged Glutathione-S-transferase in eukaryotic cell extract, then indirectly detected using anti-rat-Ig-biotin and streptavidin-POD using BM chemiluminescence western blotting substrate (POD) (https://www.sigmaaldrich.com/deepweb/assets/sigmaaldrich/product/documents/313/794/12158167001bul.pdf).
Anti-HA 3F10 Peroxidase was validated by western blot detecting for purified HA-tagged Glutathione-S-transferase in eukaryotic cell extract, then indirectly detected using anti-rat-Ig-biotin and streptavidin-POD using BM chemiluminescence western blot substrate (POD) (https://www.sigmaaldrich.com/deepweb/assets/sigmaaldrich/product/documents/348/595/12013819001.pdf).
Anti-GFP have been validated using Immunofluorescence imaging of GFP using H3-GFP construct transfected in HEK-293E cells (https://www.thermofisher.com/antibody/product/GFP-Antibody-Polyclonal/A-11122).
Anti-PGL-1 was validated in multiple publications including Strome and Wood (1983): validated by immunoprecipitation and western blot detecting for a 40KDa P-granule polypeptide in C. elegans embryo.
Anti-Myc was validated using immunofluorescence imaging of using 70% confluent His-H3-Myc transfected HEK-293 cells (https://www.thermofisher.com/antibody/product/c-Myc-Antibody-clone-9E10-Monoclonal/13-2500).
Anti-Ty1 was validated using western blot on 20 µg whole cell extracts from MCF7 cells (https://www.thermofisher.com/antibody/product/Ty1-Tag-Antibody-clone-BB2-Monoclonal/MA5-23513).
Anti-H3K9me3 and anti-H3K27me3 and have been validated in previous studies and by the manufacturer. Specifically, anti-H3K9me3 antibody was used for ChIP-seq in Ni et al., BMC 2016, and was further validated using the SNAP-CUTANA spike-in control (EpiCypher) - see Supplementary Figure 7B. Anti-H3K27me3 was validated for CUT&Tag by Kaya-Okur et al., Nature protocol, 2020.
Anti-actin IgG was validated by western blot in mammalian cell lines (NIH 3T3, MDA-MB-231, Hela) whole cell lysates, and mouse liver whole tissue lysate, then indirectly detected by goat anti-mouse IgG polyclonal (https://www.abcam.com/products/primary-antibodies/beta-actin-antibody-ab8227.html).

Animals and other research organisms

Policy information about studies involving animals; ARRIVE guidelines recommended for reporting animal research, and Sex and Gender in Research

Laboratory animals	(All animals used are young adult hermaphrodites unless otherwise noted in the figure legends)<br>C. elegans strains -<br>N2<br>YY538<br>FD53<br>TRG1656<br>JMC231<br>USC1456<br>USC1457<br>USC1464<br>USC1465<br>USC1473<br>USC1474<br>USC1475<br>USC1476<br>USC1477<br>USC1478<br>USC1479<br>USC1480<br>USC1481<br>USC1485<br>USC1486<br>USC1487<br>USC1488<br>USC1489<br>USC1492<br>USC1493<br>USC1507<br>USC1508<br>USC1510<br>USC1511<br>USC1526<br>USC1529
Wild animals	Wild animals were not used
Reporting on sex	C. elegans are hermaphrodies. Only adult animals, undergoing oogenesis but also containing sperm, were examined unless specifically noted (i.e. L4s undergoing spermatogenesis were examined in Supplementary Fig. 3A).
Field-collected samples	Field-collected samples were not used
Ethics oversight	No ethical approval is required for C. elegans.

Note that full information on the approval of the study protocol must also be provided in the manuscript.
